# Understanding and forecasting sudden explosive eruptions

**DOI:** 10.1007/s00445-025-01886-1

**Published:** 2025-10-11

**Authors:** John Stix, J. Maarten de Moor, Alessandro Aiuppa

**Affiliations:** 1 Department of Earth and Planetary Sciences, McGill University, 3450 University Street, Montreal, Quebec, H3A 0E8 Canada; 2 OVSICORI-UNA, De la UNA 200m N 100m E, Apartado 2386-3000, Heredia, 40101 Costa Rica; 3 Department of Earth and Planetary Sciences, University of New Mexico, Albuquerque, NM 87131 USA; 4 Dipartimento di Scienze della Terra e del Mare, Università degli Studi di Palermo, Via Archirafi 36, Palermo, 90123 Italy

**Keywords:** Explosive eruptions, Eruption forecast, Gas escape, Precursory signals

## Abstract

Explosive eruptions of VEI ≤ 3 commonly occur with few warning signs. Such eruptions can be magmatic, phreatomagmatic, or phreatic in nature, and they are driven by the catastrophic release of pressurized gas. Our challenge is how to better forecast these eruptions and better understand them with existing and new tools. Here we examine a number of such eruptions, some lethal to humans, which have occurred during the last decade. We first describe key precursory signals that preceded these events, examine whether they developed in a bottom-up or top-down fashion, and compare the different timescales of precursory activity. In an attempt to understand how, when, and where these systems become pressurized, we then outline the different processes and crustal locations leading to the overpressure. We further identify a number of precursory signals that may be generally applicable and exportable to such systems, and we discuss effective means of using thresholds of these precursory signals and eruptive transitions to improve our forecasting abilities. We conclude by outlining three grand challenges for the next decade: (1) complete forecasts of explosive eruptions including when, where, how big, and what type, (2) a full view of subsurface volcano plumbing, and (3) monitoring networks that are comprehensive, similar, and systematic in nature.

## Introduction

Forecasting explosive eruptions is a grand challenge in volcanology. Large explosive eruptions are commonly preceded by clear indications of unrest and instability (Punongbayan et al. 1996), and these indicators can be usefully used to assess hazard, the likelihood that the system will indeed erupt, and even the approximate timing of the eruption in some cases (Harlow et al. 1996). But many active systems, which typically experience smaller eruptions, do not show clear premonitory signs (Kato et al. 2015). In some cases, the signs are altogether absent or difficult to separate from background ambient noise, even in hindsight (Stix and de Moor 2018; Montanaro et al. 2022). Oftentimes, these volcanoes exhibit elevated and fluctuating baseline activity, complicating the identification of clear precursory signals (Joseph et al. 2022). Such volcanic systems are highly problematic from a forecasting point of view, because the explosive eruptions can occur suddenly, and if people are in the vicinity, they are at great risk. These problematic systems also demonstrate that we do not understand the full array of physical processes that are occurring in the subsurface beneath the volcano.

What is actually meant by an “eruption forecast”? Dempsey et al. (2022) define the term as the probability of an eruption occurring within a specific and/or specified time window in the future. The forecast is based upon a collection of signals or precursors that occur reliably before (most) eruptions, and that do not occur during non-eruptive unrest periods (Ardid et al. 2022). Such forecasts are most useful to the community when they can be broadly applied to many volcanoes. An eruption forecast is inherently challenging to make because sometimes a volcano is close to erupting but does not. Indeed, a volcano exhibiting unrest is reflecting a balance between forces promoting eruption (e.g., overpressure) (Blake 1984; Tait et al. 1989) and those that resist eruption (e.g., gas escape, strong roof rocks and wall rocks) (Heap et al. 2021).

In this paper, we examine six volcanic systems that have produced explosive eruptions during the past decade. These eruptions have been comparatively small, mostly VEI ≤ 3. All the eruptions were sudden and unexpected. Some of the eruptions resulted in casualties. Five of the six eruptions were not forecast because precursory signals were either absent, subtly expressed, and/or unclear in real time. Hence, the obvious challenge for future forecasting of such events is how we can improve our ability to accurately forecast and understand such sudden explosive events. Our principal motivation is to better understand an array of precursory signals with the intent of better forecasting, thus preventing or reducing future casualties.

The aim of this paper is not to produce a full review of sudden explosive eruptions. Rather, our goal is to highlight examples which we believe can substantially advance our understanding of such systems, and hence our forecasting abilities applied to these systems. In terms of approach, we focus on specific signals (e.g., gas, seismic, deformation) which we believe to be the most significant parameters to make these advances. These signals have general applicability, both for eruptions at the volcano under study and for eruptions at similar volcanoes. We fully acknowledge that the best understanding of an active volcanic system comes from gathering, analyzing, and integrating data from numerous sensors, including ground-based (Giudicepietro et al. 2019; Maeda 2023), aircraft-based, both crewed and uncrewed (Gerlach et al. 1997; Stix et al. 2018), and satellite-based (Burton et al. 2021), and augmented by statistical analysis (Bebbington 2013), artificial intelligence (e.g., Korb and Nicholson 2010; Dempsey et al. 2020; Longo et al. 2024; Ardid et al. 2025), and expert solicitation (Aspinall 2010; Bebbington et al. 2018). This multidisciplinary approach is the “best-practice” manner by which we can monitor, understand, and forecast volcanic activity (e.g., Leonard et al. 2014; Hayes et al. 2020; Poland and Anderson 2020; Christophersen et al. 2022; Rosi et al. 2022; Acocella et al. 2024). In this paper, we focus on specific signals and measurements which appear to have substantial and general utility for understanding and forecasting sudden explosive eruptions.

Here we examine four systems that exhibit a range of eruptive styles: Stromboli in Italy that produced a significant magmatic paroxysm on 3 July 2019, Poás in Costa Rica that exhibited a series of phreatomagmatic eruptions in April 2017, Whakaari in New Zealand that generated a lethal explosive eruption on 9 December 2019, and Ontake in Japan that produced another small, lethal eruption on 27 September 2014. At the time of eruption, the volcanoes were being monitored by well-established instrumentation networks. We supplement these four examples by looking at significant events that occurred prior to the 2015 phreatic eruption of Hakone in Japan, and prior to the 2021 explosive eruptions of Soufrière St. Vincent in the Lesser Antilles. Together, these six systems provide an interesting assortment of precursory signals and a basis for discussion regarding how these signals might be used to improve our future forecasting capabilities at each of these volcanoes and for other volcanic systems with similar characteristics.

Sudden explosive eruptions are all essentially driven by a catastrophic release of gas. The amount of solid material erupted is generally very small, sometimes trivial. The eruptions thus can be thought of as dominantly gas eruptions driven by accumulated and pressurized volatiles. Active volcanic systems are typically overpressured in the subsurface at one or more locations. The crucial question we seek to address in this contribution is how, when, and why a volcano can move from a state of “background” overpressure to a state of increased overpressure where the probability of a sudden explosive eruption is substantially higher. Thus the keys to understanding and forecasting these sudden eruptions are (1) identifying when and where pressurization and volatile influx/accumulation are occurring, (2) assessing how much pressure/gas is accumulated and how suddenly and when it will be released, (3) understanding the physics and mechanisms of seal failure, and (4) identifying the precursors in real time before the eruption takes place.

Hence, the principal goal of this paper is to understand how the behavior and processes of an active volcanic system prior to an explosive eruption, or sequence of eruptions, relate to our ability to forecast such events. We first assess various precursory signals associated with the significant eruptions from the four systems studied here. We then examine subsurface processes using both a bottom-up and top-down perspective, and we assess the different timescales of precursory activity in terms of forecasting capabilities. Following this, we address different mechanisms that generate overpressure, endeavoring to identify where and when gas pressure may be building. Then, we discuss the issue of precursors, thresholds, and eruptive transitions that might be applied reliably to other volcanoes. We conclude with two sections that discuss where we are headed during the next decade.

## Precursory signals

### The 3 July 2019 eruption of Stromboli (Italy) (VEI ≤ 3)

Stromboli is a highly active volcano in the Aeolian Islands north of Sicily. It is both a basaltic and explosive system with a range of eruptive styles. Typical activity comprises low-level Strombolian explosions on timescales of minutes to hours. Beyond this persistent low-level activity, the volcano can produce more powerful and dangerous eruptions (Rosi et al. 2013; Pioli et al. 2014) that are classified as “major” to “paroxysmal” explosions. Major explosions typically occur once or twice a year, while the largest paroxysmal events (violent and short-lived eruptions) typically occur once or twice each decade (Bevilacqua et al. 2020). Such paroxysmal events have eruption columns extending to several kilometers in altitude, associated pyroclastic density currents, and sometimes flank collapse with tsunami generation. There have been 36 paroxysms in the last 140 years, with an average recurrence interval of 3.9 years (Bevilacqua et al. 2020).

**Fig. 1:**
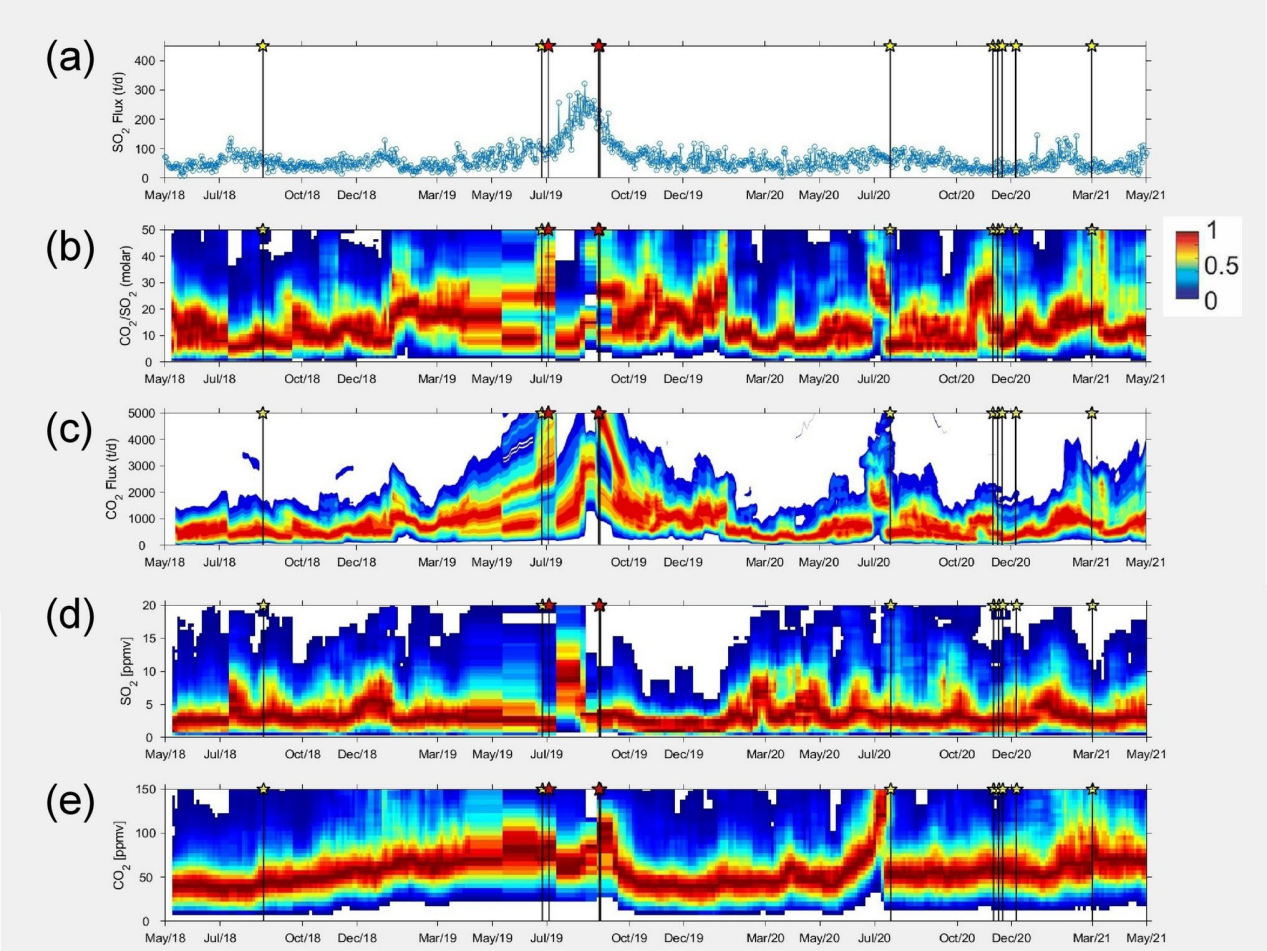
Gas parameters for Stromboli, 2018–2021, modified from Aiuppa et al. (2021). Red stars indicate paroxysms, yellow stars indicate major explosions. **a** SO_2_ fluxes in metric tons per day. **b** Molar CO_2_/SO_2_ ratios. **c** CO_2_ fluxes in metric tons per day. **d** SO_2_ concentrations in parts per million per volume (ppmv). **e** CO_2_ concentrations in ppmv corrected for atmospheric background. The temporal plots illustrate the normalized (scale 0 to 1) frequency distribution diagrams of all parameters, in which dark red tones indicate the dominant concentration/ratio levels, while orange to blue tones indicate increasingly less represented frequency bins. Increases in CO_2_/SO_2_, CO_2_ flux, and CO_2_ concentrations occurred over various timescales before the 3 July 2019 paroxysm. These parameters are also commonly elevated prior to some major explosions in 2020–2021. SO_2_ fluxes and CO_2_ fluxes increased before the 28 August 2019 paroxysm

In the summer of 2019, two paroxysms occurred, the first on 3 July and the second on 28 August. Neither of the two eruptions were forecast in strict terms, but the second was identified in hindsight based on increasing gas fluxes before the event (Fig. 1; Aiuppa et al. 2021). The 3 July event was among the largest instrumentally monitored paroxysms, i.e., it had among the highest tilt signals on record in the past two decades (Ripepe et al. 2021) and was preceded by a major explosion one week prior on 25 June (Métrich et al. 2021). The 3 July paroxysm was responsible for the death of one hiker and caused closure of the summit area to tourists (still ongoing at the time of writing). Giudicepietro et al. (2020) suggested that the system began a new phase of activity in May 2017, with nine major explosions from July 2017 to August 2018. Aiuppa et al. (2021) examined SO_2_ and CO_2_ data from the summit MultiGAS station, showing that CO_2_ concentrations began to slowly rise starting in late July or early August 2018, nearly one year prior to the eruption (Fig. 1e). In the last half of 2018, mean CO_2_ concentrations were generally less than 50 ppm above background concentrations (420 ppm), but by December the levels were consistently exceeding 50 ppm. Maximum CO_2_ concentrations were less than 75 ppm until November 2018; thereafter they commonly exceeded 75 ppm and sometimes reached 100 ppm or even higher, particularly after January 2019 (Fig. 1e).

Mean CO_2_/SO_2_ ratios in 2018 were lower than 15, then exhibited a large increase in early 2019, with values exceeding 15 thereafter. Immediately prior to the 3 July eruption, ratios were bimodal, with low values of ~ 10 and high values of 20–40. Immediately after the eruption, mean ratios were < 10 and no longer bimodal (Fig. 1b).

Two campaigns of carbon isotope measurements on plume gases were carried out, the first in May 2018 and the second in late June 2019, two weeks before the 3 July eruption (D’Arcy et al. 2024). The surveys used drones to sample gas from the different vents, and same-day carbon isotope analyses were made on the samples using Picarro and Delta Ray instrumentation. In 2018, the CO_2_ δ^13^C signature of the plume was − 0.36 ± 0.59‰, while in 2019 it was − 5.01 ± 0.56‰, a shift of nearly 5‰ between the two campaigns.

One month prior to the 3 July eruption, the amplitude of very long period (VLP) seismic events began to increase significantly above baseline levels of < 1000 (digital counts), with values reaching 2000 or more for about a week immediately prior to the eruption (Giudicepietro et al. 2020). These authors also documented a late-stage strain decrease on 3 July at the SVO station near the town of Stromboli lasting for about an hour that was immediately followed by a large strain increase 11 min before the eruption. This strain increase corresponds with rapid inflation of 10–14 μrad of the summit region during the 12 min prior to the eruption (Ripepe et al. 2021).

In summary, unusual gas signatures were observed well before the eruption, with CO_2_ concentrations starting to increase nearly a year beforehand, followed by increasing CO_2_/SO_2_ about six months prior. These changes are consistent with the introduction of either CO_2_-rich gas bubbles or volatile-rich magma accompanied by CO_2_ exsolution from this new magma into the deep plumbing system at 6–9 km (Métrich et al. 2021), as revealed by the carbon isotopic data (D’Arcy et al. 2024). The anomalous VLP seismicity observed in June, 1 month before the eruption (Giudicepietro et al. 2020), is likely a manifestation of a gas-charged and CO_2_-rich pressurized system that was primed to erupt. The changes in strain and inflation that occurred immediately prior to the eruption were likely the result of the rapid rise of buoyant magma from 6 to 7 km depth to the surface.

### The April 2017 eruptions of Poás (Costa Rica) (VEI 2)

Poás is historically the most active volcano in Costa Rica and hosts an important hydrothermal system including an acid crater lake. Unrest and eruptions are frequent, and the volcano exhibits multiple eruption styles, including phreatic, phreatomagmatic, and magmatic eruptions. The volcano sits within a complex transtensional fault zone (Montero et al. 2010; Stix et al. 2025), and the volcano is part of a larger caldera structure or structures (Prosser and Carr 1987).

From 13 to 23 April 2017, a series of phreatomagmatic eruptions occurred, the largest since the mid-1950s, with columns up to 4 km. Various changes in activity were observed in near real time prior to the eruptions, including minor phreatic events, increased seismicity, and increased degassing. The national park was closed on 9 April due to strong degassing that impacted areas visited by tourists, yet no formal forecast was issued prior to the most violent eruption on 14 April, when bombs impacted the overlook platform and damaged infrastructure. Although increased eruptive activity was “anticipated,” the timing and magnitude of this eruption were not foreseen or forecast.

**Fig. 2:**
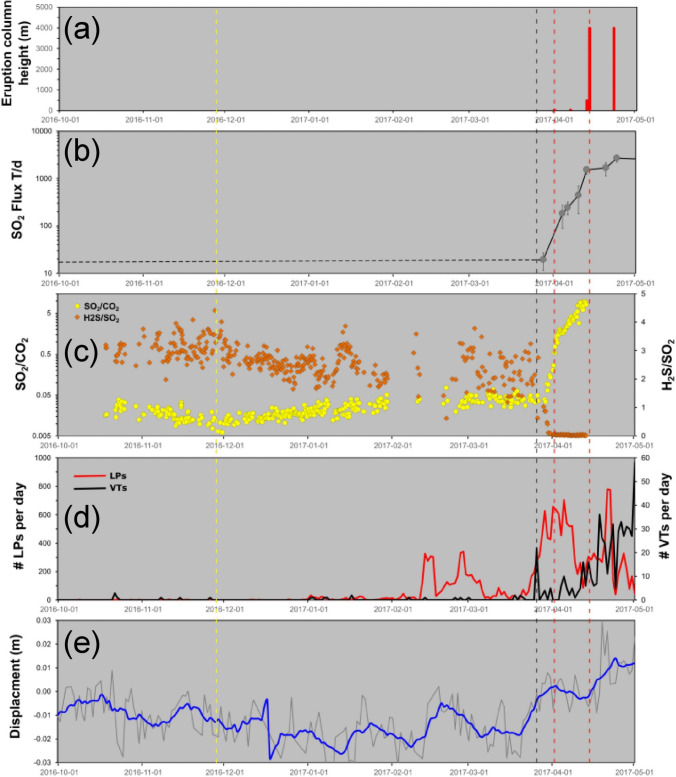
Gas and geophysical parameters for Poás, 2016–2017, modified from de Moor et al. (2019). **a** Eruption column heights in meters above the summit. **b** SO_2_ fluxes in metric tons per day. **c** Molar SO_2_/CO_2_ ratios. **d** Number of daily earthquakes; red line is long period (LP) events, black line is volcanotectonic (VT) events. **e** Vertical displacement in meters, as measured at station VPCR; the light gray line is raw data while the blue line is a 7-day moving average. Subtle changes in SO_2_/CO_2_ and H_2_S/SO_2_ began occurring in late November 2016 (vertical dashed yellow line), characterized by a change in slope of both parameters. Large changes in SO_2_ fluxes and gas ratios were observed after the 26 March 2017 volcanotectonic seismic swarm (vertical dashed black line), Long-period seismicity began to be noticeable in February 2017, while ground deformation showed no obvious changes until March–April 2017. Vertical dashed red lines indicate the first small phreatic eruption (eruption column ~ 50 m) and the first violent explosive eruption (eruption column ~ 4000 m)

In late 2016 and early 2017, the shallow plumbing system beneath the volcano exhibited a clear hydrothermal signature, with gases emitted at the surface characterized by SO_2_/CO_2_ less than 0.03, H_2_S/SO_2_ at 2–4, and SO_2_ fluxes < 50 metric tons per day (t day^−1^), suggesting that the system was strongly sealed at shallow levels due to sulfur scrubbing processes and clogging of degassing pathways by hydrothermal mineral precipitation (de Moor et al. 2019) (Fig. 2). In late October – early November 2016, the gas ratios observed at the lake MultiGAS station started to shift, with SO_2_/CO_2_ increasing and H_2_S/SO_2_ decreasing by small amounts. Long period (LP) seismicity appeared in early January 2017 and then increased significantly in early to mid February, with > 300 LP events/day occurring at times, before declining to < 100 events/day in March (Salvage et al. 2018). This was followed by observable inflation beginning in mid-March. On 26 March, a volcanotectonic (VT) earthquake swarm was recorded, jumpstarting the eruption sequence. Immediately after the swarm, LP events increased dramatically to > 600 events/day (Fig. 2d), but tremor and RSAM did not increase significantly until after the initiation of the eruptive sequence (Salvage et al. 2018). Volcanic gases showed similarly large changes, with SO_2_/CO_2_ increasing from < 0.07 in mid to late March, to 0.1 on 30 March, 0.44 on 1 April, and > 5 after 9 April, five days before the most significant eruption on 14 April. Hence, SO_2_/CO_2_ increased by two orders of magnitude before this potentially fatal eruption. During the same time, H_2_S/SO_2_ fell from values of 2–4 to essentially zero, while SO_2_ fluxes increased from 20–30 t day^−1^ to > 1000 t day^−1^ on 13 April (de Moor et al. 2019) (Fig. 2b). Stable isotopes of CO_2_ in fumarolic gases also shifted substantially toward more negative values, from − 4.2‰ on 24 February 2017 to − 6.2‰ on 6 April about 1.5 months later (D’Arcy et al. 2022).

To summarize, the subtle changes in gas ratios beginning in late 2016 are suggestive of small, early “leaks” of magmatic fluid through the otherwise well-sealed hydrothermal system. The large increases in LP seismic events in February 2017 could indicate significant pressurization of the system below the hydrothermal seal. The VT seismic swarm on 26 March 2017 was likely due to fracturing of the hydrothermal seal, providing new pathways for magmatic fluids to the surface and, in effect, initiating the eruption sequence 18 days later. The correlated changes in gas compositions and ratios in late March and early April are indicative of a system that rapidly switched its behavior from nearly purely hydrothermal to nearly purely magmatic during the course of several weeks’ time.

### The 9 December 2019 eruption of Whakaari (New Zealand) (VEI 2)

Whakaari, also known as White Island, is New Zealand’s most active volcano. It is a mostly submarine edifice, with the upper 321 m projecting above sea level. The volcano has a highly active hydrothermal system, with magma and hydrothermal fluids dynamically interacting at shallow levels beneath the volcano, which also frequently hosts an acid lake. Styles of eruptive activity are varied and diverse, with lava dome eruptions as well as explosive activity, including magmatic strombolian, phreatomagmatic, and phreatic (Kilgour et al. 2021).

**Fig. 3:**
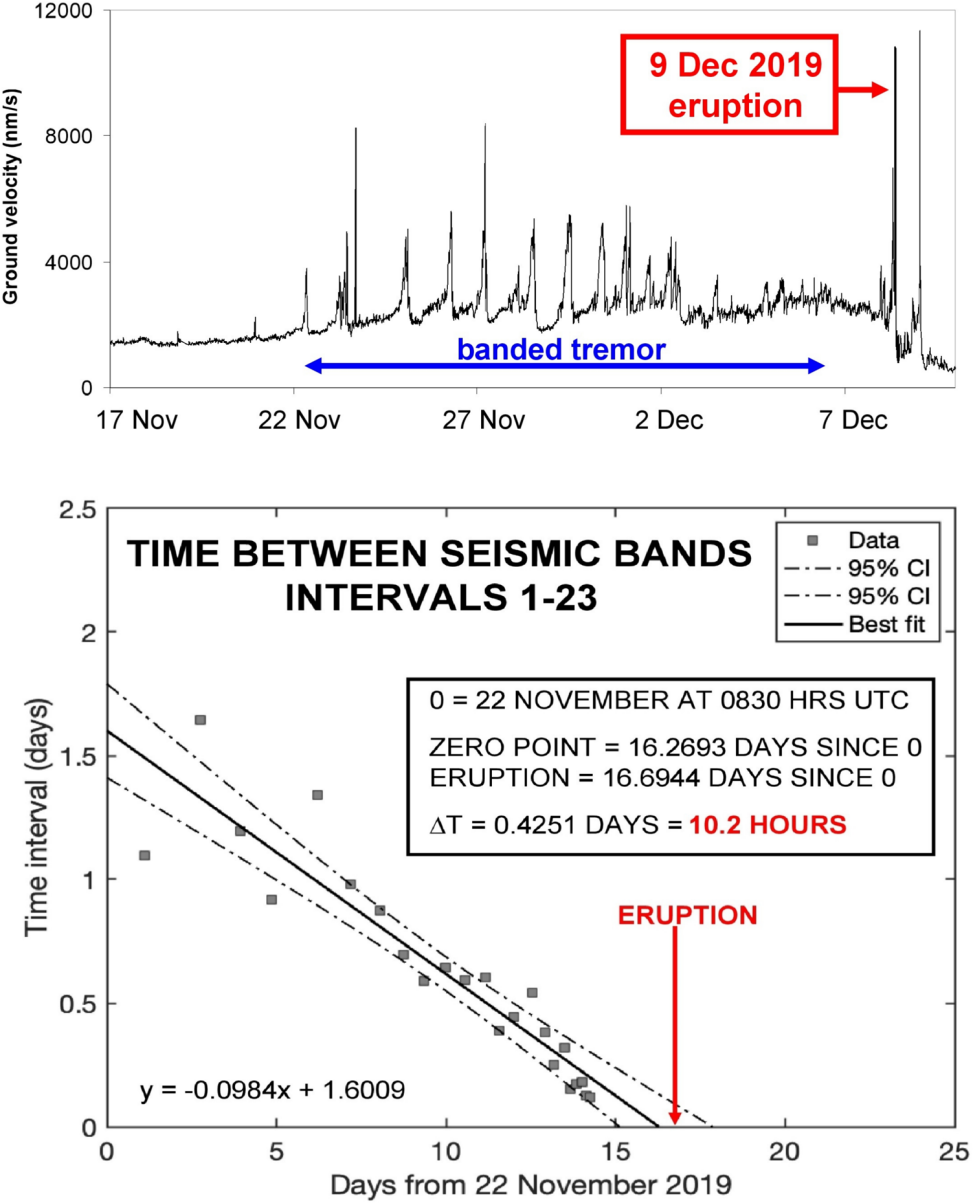
Seismic data for Whakaari during November–December 2019. The top panel shows ground velocity recorded by the WSRZ seismic station at Whakaari from 17 November to 10 December 2019. Data are 10-min, 2–5 Hz-filtered RSAM. The raw seismic data are from GNS Science (2021). Banded tremor is well displayed from 22 November to 2 December and less pronounced from 2 to 6 December. The bottom panel shows systematically decreasing intervals between tremor bands. By linearly extrapolating these decreasing intervals to a value of zero (the “zero point”), it is possible to make a hindcast estimate of the 9 December eruption to within 10.2 h. From Stix et al. (2024)

Beginning in a 2012, a series of small explosive phreatic and phreatomagmatic eruptions have occurred, including one in 2012, two in 2013, one in 2016, one in 2019, and one in 2024. The 2019 eruption, which occurred on 9 December at 1411 h local time (0111 h UTC), killed and injured a number of people who were visiting the crater at the time. Prior to this eruption, a series of unusual tremor signals began to occur on 22 November and were observable until 7 December (Stix et al. 2024) (Fig. 3). The signals may have actually begun on 18 November, although there is some uncertainty here. Thus, the tremor sequence began 17–21 days before the eruption. The tremor was characterized by a series of regularly repeating peaks and troughs, termed banded tremor, with amplitudes of tremor peaks increasing rapidly to comparatively high levels and then maintained until 2 December when the peak amplitudes decreased. A further decline in peak amplitudes occurred on 6 December. This relative seismic quiet was maintained until two large tremor spikes were recorded 17.5 and 16.2 h before the eruption. After the eruption, tremor was at a low level for about a day before ramping up to very high levels during the following three days, accompanied by high rates of SO_2_ degassing (GeoNet 2019a).

The most interesting aspect of this banded tremor sequence is the time interval between tremor peaks. Plotting these intervals as a function of elapsed time from the initial tremor peak reveals a systematic, quasi-linear decrease in the intervals. Linearly regressing the data and calculating the “zero point,” i.e., the theoretical point in time when the intervals become zero (y = 0), provides an accurate hindcast within 10.2 h of when the eruption would occur (Fig. 3). A similar analysis of tremor signals before the 27 April 2016 eruption provides an even more accurate hindcast to within 3–5 h of the eruption (Stix et al. 2024).

In summary, the striking banded pattern in the tremor sequence at Whakaari provides not only an accurate hindcast but also allows us to infer subsurface processes. A model described by Stix et al. (2024) involves the injection of hot magmatic fluids into the hydrothermal system on or about 18–22 November, pressurizing the system and causing the hydrothermal fluids to boil periodically. Progressive magmatic heating and pressurization caused the fluids to boil more frequently, accounting for (a) the decreased intervals between tremor peaks and (b) the increasing peak amplitudes from 18–22 November to 27 November. The decreased amplitudes thereafter, as well as the seismic quieting on 2 December, may reflect the fact that the core of the hydrothermal system had been largely converted from liquid and/or two-phase fluid to a state of high pressure where vapor was dominant (Stix et al. 2024). Importantly, the occurrence of the banded tremor, coupled with the decreasing intervals between tremor peaks, provides us with a key clue that an explosive eruption was imminent.

### The 27 September 2014 eruption of Ontake (Japan) (VEI 3)

Ontake is a volcano located in central Honshu island, Japan. The volcano was dormant until an explosive eruption in 1979, the first in recorded history. Since then, there have been small explosive eruptions in 1991, 2007, and 2014 (Nakamichi et al. 2009; Yamaoka et al. 2016). None of these eruptions were forecast, and the last event claimed the lives of at least 58 people who were near the summit at the time of the eruption. This was the worst volcanic disaster Japan since the eruption of Mt. Tokachidake in 1926, and this catastrophic event stimulated many studies to retrospectively examine the precursors and causes of this small but deadly eruption (Yamaoka et al. 2016).

**Fig. 4:**
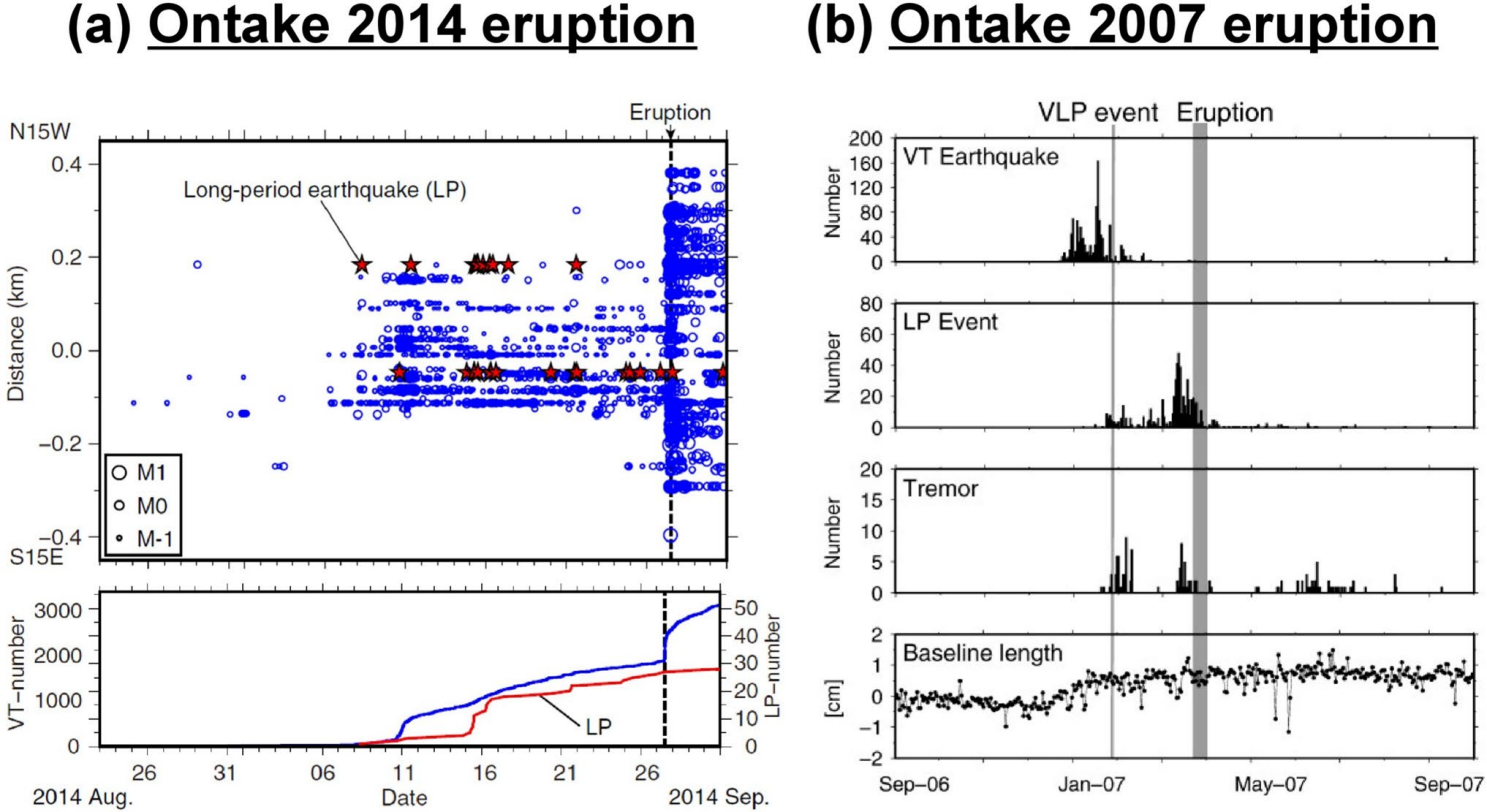
**a** LP and VT seismic events beneath Ontake volcano in August–September 2014. The upper panel shows the different events, and the lower panel shows cumulative events. Blue circles are VT events, and red stars are LP events. Note the sudden increase in both LPs and VTs on or about 6 September, three weeks before the eruption on 27 September. From Kato et al. (2015). **b** Seismicity and deformation prior to the late March 2007 eruption of Ontake. The upper three panels show seismicity, and the lower panel shows GNSS displacements. The thin vertical grey line shows when the VLP signal occurred on 25 January 2007, while the thick vertical grey line shows the approximate timing of the eruption (the exact date and time are not known). Note the simultaneous occurrence of VT events and inflation during December 2006 and January 2007, followed closely by the VLP signal, LP events, and tremor starting in late January. Also note the increased LP events in the weeks immediately before the eruption. From Nakamichi et al. (2009)

A series of subtle seismic and geodetic signals occurred before the eruption. A sudden increase in both VT and LP events occurred around 6 September 2014, three weeks before the eruption on 27 September. A total of ~ 3000 VT and ~ 28 LP events were recorded during this three-week window (Kato et al. 2015) (Fig. 4a). The *b*-values of the VT events showed an initial increase, peaking at a value of ~ 1.7 in mid-September, then declining to a value of ~ 0.8 about three days before the eruption (Kato et al. 2015). On a longer timescale, Caudron et al. (2022a) recognized a seismic velocity decrease beginning about 3 months before the eruption, and also noted a three-day period of tremor in mid-August. GNSS data from the Japanese Meteorological Agency showed remarkable stability since 2007 (Yamaoka et al. 2016). However, a re-analysis of the data using a stacked technique to reduce noise revealed subtle inflation from 1 to 1.5 months prior to the eruption (Miyaoka and Takagi 2016).

After the eruption, SO_2_ fluxes were elevated at several hundred to several thousand tons per day, but by late November, SO_2_ fluxes had fallen to 130–140 t/d (Mori et al. 2016). H_2_S/SO_2_ molar ratios increased from 3.2 on 9 October 2014 to 10.3–10.8 in late November and to 29.4 in June 2015 (Mori et al. 2016).

The elevated SO_2_ fluxes and relatively low H_2_S/SO_2_ directly after the eruption suggest that magmatic gas was being expelled from the system, and this had largely stopped by November 2014 (Mori et al. 2016). Hence, the seismic and geodetic unrest noted in August–September 2014 prior to the eruption is likely the manifestation of magmatic gas injection into the shallow hydrothermal system (Kato et al. 2015; Mori et al. 2016). Sano et al. (2015) observed a long-term decadal increase in ^3^He/^4^He at the Nigorigo hot spring closest to the summit, while Terakawa (2017) mapped a source of overpressured fluids at 7.5 km depth beneath the east flank of the volcano, which may have channeled magmatic gas into the hydrothermal system.

Despite this magmatic input, appreciable amounts of H_2_S were emitted by the volcano after the eruption, and the H_2_S/SO_2_ ratio of 3.2 measured on 9 October 2014 attests to a dominantly hydrothermal gas (Mori et al. 2016). Sulfur isotopes measured in sulfur, sulfates, and sulfides from ash collected after the eruption indicate disproportionation of SO_2_ into the hydrothermal system (scrubbing) (Ikehata and Maruoka 2016), consistent with a mixture of hydrothermal and magmatic gas emitted after the eruption.

To summarize, Ontake exemplifies the very difficult problem of forecasting a system whose precursory signals are few and subtle. Despite the pre-eruptive quiet state of this volcano, the 2014 eruption demonstrates that the system was overpressured and capable of erupting. It is not fully clear whether the eruption was phreatic or phreatomagmatic. Miyagi et al. (2020) examined ash from the 2014 eruption and documented the presence of less altered glassy material, which could represent magma injected into the system in 2007.

At Ontake, a VLP event occurred 25 s prior to the 2014 eruption, indicating crack propagation and fluid transport to the surface (Maeda et al. 2015). In 2007, a VLP signal was detected on 25 January about 2 months before the eruption in late March (Nakamichi et al. 2009). This VLP event was preceded by VT seismicity and small amounts of inflation lasting 1 to 2 months. Tremor and LP seismicity suddenly began several days before the VLP event and continued until the eruption. The LP seismicity increased for a month before the eruption, then declined to very low levels afterward (Nakamichi et al. 2009) (Fig. 4b). This sequence of events may reflect the injection of magma and/or magmatic fluids into the shallow hydrothermal system beneath Ontake, causing it to pressurize, as reflected by the VLP, tremor, and increasing LP signals prior to the eruption. It has been suggested that the VLP signal was generated by transient crack formation accompanied by the flow of vaporized fluid (Nakamichi et al. 2009). In contrast to 2014, the crack did not reach the surface.

In cases such as Ontake, where unrest is absent or low-level, a sudden increase in activity, even if small, is potentially highly significant, as it may be revealing that the volcano is capable of erupting and may in fact erupt. The simultaneous appearance of VT and LP seismicity three weeks before the 2014 eruption, coupled with the high SO_2_ levels observed soon afterward, is a clear indication, in retrospect, that magmatic fluids were being injected into the shallow hydrothermal system, causing it to pressurize and then erupt. Likewise, prior to the 2007 eruption, the sudden and simultaneous appearance of VLP, LP, and tremor signals approximately 2 months prior to the eruption is a clear indication, in retrospect, of the shallow hydrothermal system progressively pressurizing, which ultimately caused the volcano to erupt.

## Bottom-up vs. top-down

The types of volcanic systems discussed here have complex plumbing extending from the surface to the mantle. Typically, we have a small window of insight into a restricted section or sections of the volcanic plumbing systems. Geological, geophysical, and geochemical imaging allows us to map some of this plumbing, but a detailed picture of the full systems is elusive. How plumbing systems change with time is crucial to understanding the physics of these highly dynamic systems and a challenge for the next decade.

Despite these limitations, we can examine processes that may occur at deep, intermediate, and shallow levels. In the case of Stromboli, paroxysms are the result of both shallow and deep processes. Sometimes, rapid effusion of lava occurs at the summit, causing depressurization of the deeper system and an eventual paroxysm, as exemplified by the March 2007 and August 2019 events. By contrast, the July 2019 paroxysm was the result of inflow of gas or gas-rich magma into the deep plumbing system at 6–9 km depth, with concomitant CO_2_ exsolution, and this process appeared to begin nearly a year before the eruption (and possibly even longer) as shown by the early increases in CO_2_ concentrations recorded by the summit Multi-GAS station (Fig. 1e; Aiuppa et al. 2021). Hence, these deep processes appear to operate on relatively long timescales of months to years.

At Poás, evidence for shallow processes is abundant. A hydrothermal seal likely existed in late 2016 and early 2017, as attested by the very low SO_2_/CO_2_ and very high H_2_S/SO_2_ (Fig. 2). de Moor et al. (2019) interpreted the gas compositions and low SO_2_ fluxes to reflect SO_2_ scrubbing processes resulting in precipitation of hydrothermal minerals that resulted in decreased vent permeability and clogged degassing pathways. An interesting question concerns the strength and quality of the seal, as well as the reason for and timing of its failure. Both gas ratios referred to above show some degree of variability during the sealed period, suggesting that the seal may have been leaky to some extent, allowing small amounts of magmatic gas to reach the surface, mixing with the predominantly hydrothermal gases generated in the overlying mostly sealed conduit. Exactly how and why this leaking started is unclear, and whether these changes were in response to changes in the deeper magmatic system is an open question. Nevertheless, the seal appears to have maintained its coherence until the VT seismic swarm on 26 March 2017. At this point, the seal suddenly failed as the pressure of accumulated magmatic gas beneath the seal exceeded the rock strength of the clogged conduit. Fracturing increased the permeability of the conduit, channeling large amounts of magmatic gas and small amounts of magma to the surface. Deeper-level processes appear to be subordinate here. There is no clear evidence for injection of magma from deep levels early in the runup to eruption, based on three lines of evidence. First, the magma was andesitic in composition and more evolved than the basalt erupted in the 1950 s, as evidenced by plastic juvenile bombs ejected on 22 April. Second, eruptive SO_2_/CO_2_ ratios were very high, implying a shallow source. Third, the observed pre-eruptive deformation was of small magnitude and occurred at a late stage. However, a fundamental issue in this system is the limits of detection of changes in the magmatic system beneath the hydrothermal system, which modifies signals from the deeper plumbing system. The time-averaged SO_2_ flux at Poás is in the range of 100–200 t day^−1^, comparable to Stromboli, pointing to the fact that sustained magma movement drives the system. Did the hydrothermal seal form in response to a short-term decrease in magmatic supply or in response to fundamental changes in the hydrothermal system?

At Whakaari, there is a strong coupling between the magmatic system and the hydrothermal system at shallow levels (< 1 km). There are undoubtedly deeper processes at work beneath the volcano, but the geophysical signals that are recorded, such as tremor, are likely to be the result of shallow processes (Stix et al. 2024). Yet at these shallow levels, bottom-up and top-down processes can be discerned. The observed tremor sequence before the 9 December 2019 eruption is most reasonably ascribed to the injection of magmatic fluids and heat into the hydrothermal system, causing it to boil periodically. The hydrothermal system was sealed at its top, allowing pressure to build below the seal. Was the seal leaky prior to the eruption? The evidence is inconclusive. Sporadic TROPOMI measurements recorded SO_2_ fluxes lower than 350 t day^−1^ (Burton et al. 2021), while visual observations recorded periodic gas venting at the surface (GeoNet 2019b). Yet the seal must have been sufficiently complete and coherent in order to produce sufficient overpressure as the main driver for the eruption. The large tremor spikes observed 17.5 and 16.2 h prior to the eruption indicate that the seal began to weaken and fail at this time (Stix et al. 2024), leading to the subsequent eruption.

A similar situation likely existed at Ontake prior to the 2014 eruption. Magmatic fluids were injected from deeper levels into the well-sealed shallow hydrothermal system. As a result, the system was pressurized sufficiently to generate an eruption. The same general process also occurred prior to the 2007 eruption. Although the direct involvement of magma is difficult to ascertain in both cases, the presence of magmatic fluids as drivers of overpressure is clear. This magmatic fluid input may have been occurring on two timescales, the first slow over a decade as suggested by the helium isotope data (Sano et al. 2015), and the second fast over a period of weeks to possibly months as suggested by the seismic data (Kato et al. 2015; Caudron et al. 2022a).

## Timescales of precursory activity

The four examples of sudden eruptions examined here all exhibit precursory signals at different timescales. At Stromboli, changes in gas composition occurred over periods of 6–12 months (Aiuppa et al. 2021). By contrast, measureable tilt changes were initiated ~ 12 min before the 3 July 2019 eruption (Ripepe et al. 2021). The amplitudes of the VLP seismic events began to increase 1 month before the eruption (Giudicepietro et al. 2020). Similarly, at Poás, subtle changes in gas ratios began occurring 5–6 months before the eruptions, and large, rapid, and unambiguous changes in the gas ratios were observed in the two weeks leading up to the eruption. Long-period seismic events became significant 2 months beforehand, while inflation was observed 1 month prior to the eruptions, and tremor only ramped up after the initiation of the eruptive sequence. At Whakaari, the banded tremor sequence began about three weeks before the eruption, followed by seismic quieting one week beforehand and the initial failure of the hydrothermal seal 17.5 h before the eruption. Longer preparatory timescales at Whakaari may be manifested by the explosive eruptions since 2012, which typically occur every few years. For the 2007 and 2014 Ontake eruptions, small changes in seismicity were noted 2–3 months beforehand, followed by subtle yet significant accelerations in activity in the weeks before the eruptions.

The subtle precursory signals outlined above were largely recognized in hindsight. Difficult questions include the following. (1) When do these changes become so evident that we can use them to send out an alert? (2) Do these changes consistently occur before all eruptions, and can they occur when no eruption ensues? We also note that different geochemical or geophysical trends can occur at different volcanoes depending on the processes involved. By comparing gas data, for example, at Stromboli the eruption was preceded by increasing CO_2_/SO_2_, whereas at Poás, decreasing CO_2_/SO_2_ (or increasing SO_2_/CO_2_) preceded the eruption.

**Fig. 5:**
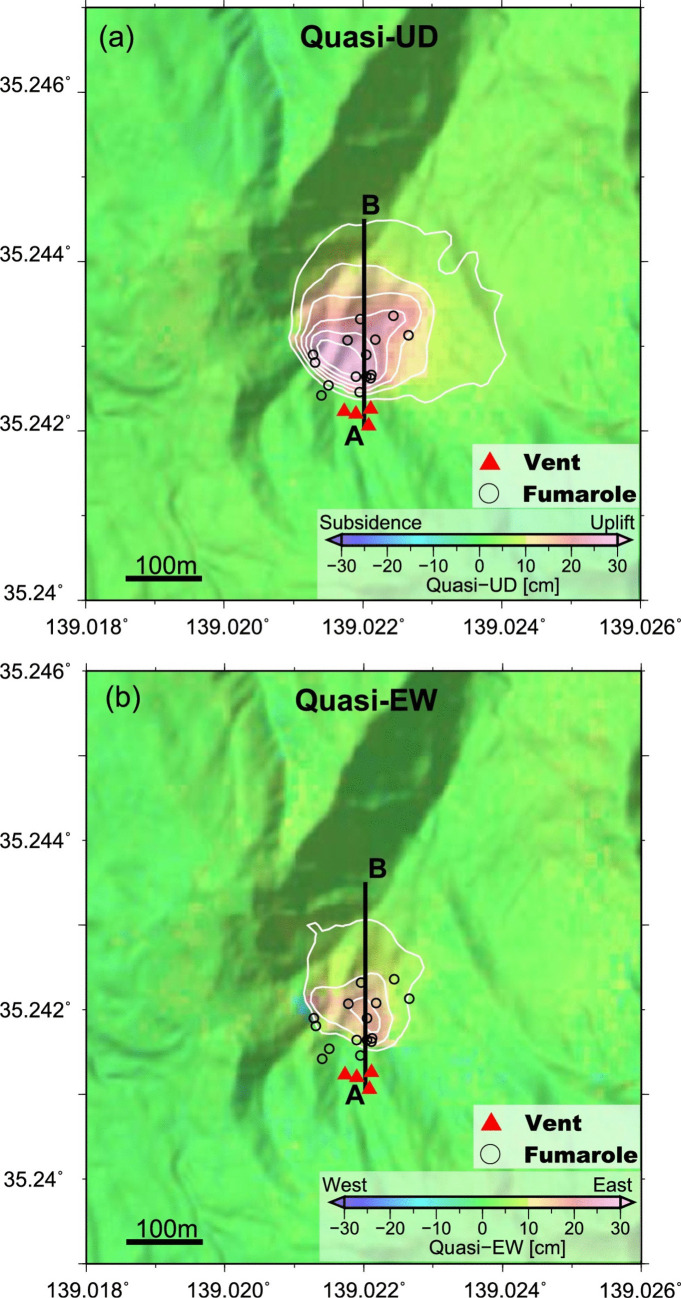
Cumulative ground displacements from ALOS-2 data at Hakone volcano, Japan, from early May to mid-June 2015. **a** Quasi-up-down displacements. **b** Quasi-east–west displacements. Note the very high spatial resolution of the data, the asymmetric ground displacements, and the close correspondence between maximum displacement and eruptive vent locations. From Kobayashi et al. (2018)

Two additional systems illustrate important precursory behavior. Hakone volcano Japan experienced a small phreatic eruption on 29–30 June 2015, its first in 700–800 years. Soufrière St. Vincent Lesser Antilles underwent a series of powerful magmatic explosive eruptions in April 2021. At Hakone, subtle inflation began occurring about a year before the eruption across two GNSS stations located outside the caldera. The inflation accelerated during April–June 2015, with a change of ~ 2 cm during this period (Kobayashi et al. 2018). Thus, two timescales of inflation can be clearly observed. InSAR measurements at very high spatial resolution reveal a highly localized inflation anomaly that appeared 6–7 months before the eruption, with significant acceleration during May–June 2015, causing uplift up to ~ 30 cm in a spatially restricted area of 100–200 m diameter (Fig. 5). Thus, the InSAR data also exhibit two timescales of deformation. The May–June uplift was strongly asymmetric, with maximum values in the south-southwest sectors, and the maximum uplift occurred several tens of meters from the eventual eruptive vents (Kobayashi et al. 2018) (Fig. 5). Thus, the InSAR data can be used to hindcast the location of the vents.

**Fig. 6:**
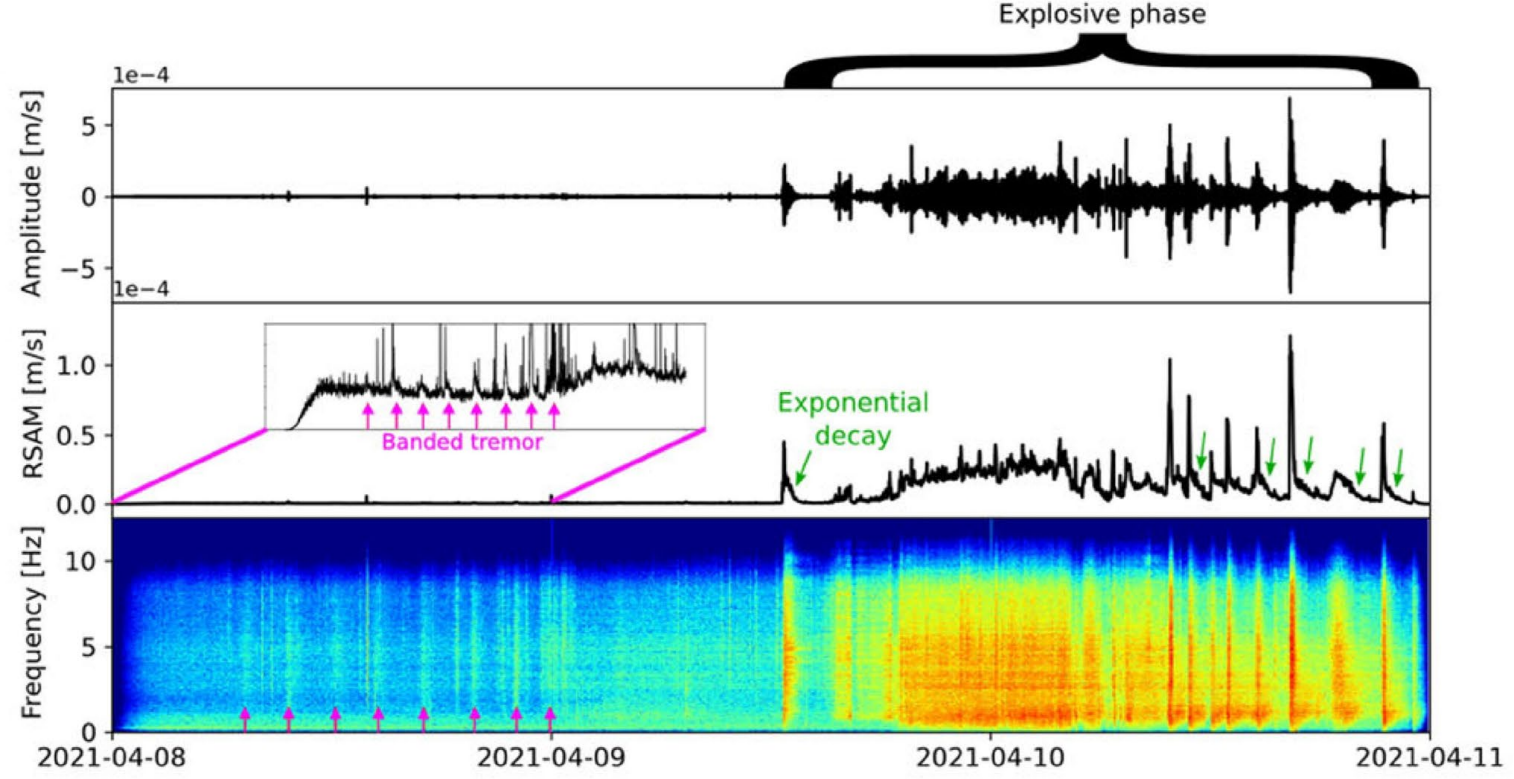
Banded tremor observed at Soufrière St. Vincent, Lesser Antilles, on 8 April 2021, one day before explosive eruptions commenced. There are eight tremor bands that began around noon UTC, roughly 1 day prior to the first explosive eruption at 12:41 h UTC on 9 April. The spacing between the tremor bands is about 2.5 h. From Joseph et al. (2022)

At Soufrière St. Vincent, a lava dome grew at a quasi-linear rate of 1.8 m^3^ s^−1^ from late December 2020 to early April 2021, associated with generally increasing LP seismicity during this time (Contreras-Arratia et al. 2024). Significant VT earthquake swarms were recorded on 23–24 March and 5–6 April 2021 (Joseph et al. 2022). A subtle increase in the RSAM baseline was registered beginning at about 0700 h UTC on 7 April (UWI Seismic Research Centre 2021a). Dome growth accelerated by an order of magnitude up to 17.5 m^3^ s^−1^ starting 15–39 h before the first explosive eruption on 9 April (Dualeh et al. 2023). SO_2_ emissions began to increase ~ 35 h prior to the explosive events (Esse et al. 2024), while banded tremor began occurring ~ 30 h beforehand (Joseph et al. 2022) (Fig. 6). On an RSAM plot, the seven tremor bands reveal generally increasing amplitudes with time (UWI Seismic Research Centre 2021b). Thus, at Soufrière St. Vincent, two timescales can be seen: steady lava dome growth over slightly more than 3 months and late-stage changes occurring rapidly over the space of a day or so immediately prior to the explosive sequence.

The longer-term timescales over months to about a year seen at these systems indicate an early and extended period of precursory and preparatory behavior, which is also true for many systems that do not erupt (Moran et al. 2011). For these non-erupting systems, it is not well understood whether the volcano was close to erupting or not, but it demonstrates that a non-eruptive system has the potential to erupt, which is important in terms of hazard analysis. However, these occurrences of potential precursors without eruption complicate forecasting efforts, as false alerts can have negative effects on the public and decrease the credibility of hazard assessment agencies.

Immediately prior to the explosive eruption, rapid short-term changes (hours to days) typically superpose themselves on top of the more subtle long-term changes. Specific types of unrest at these late stages can trigger or facilitate the principal eruption. At Stromboli, the 25 June 2019 major explosion may have fractured and weakened the plumbing system, leading to the 3 July eruption. At Poás, the 26 March 2017 VT seismic swarm likely reflects the opening of fractures permitting passage of magmatic fluids and then magma to the surface, culminating in the 13–23 April eruptions. At Soufrière St. Vincent, the 5–6 April 2021 VT seismic swarm was quickly followed by banded tremor, increased SO_2_ emissions, and accelerated dome growth on 8 April, and explosive eruptions began on 9 April. At Whakaari, the amplitudes of the banded tremor decreased appreciably on 2 December (Fig. 3), leading to a week of comparative seismic quiet before the hydrothermal seal began to fail catastrophically 17.5 h before the 9 December eruption. Under these conditions, the forecasting window is short, typically on the order of days. Banded tremor, as seen at Whakaari and Soufrière St. Vincent, is a key clue to the proximity of an explosive eruption (Joseph et al. 2022; Stix et al. 2024).

## Gas accumulation, overpressure, and sealing

The longer-term timescales illustrated above suggest that the precursory stages of an eruption can occur on a continual basis, sometimes developing at low levels that are not much above the background state of the system and that may not be evident or obvious at the time. These long timescale signals imply that processes such as magmatic inputs, sealing rates, and overpressurization can evolve slowly and steadily. A further implication is that the magmatic-hydrothermal plumbing system distributed through the crust is at times interconnected (Cashman et al. 2017), with one component of the system influencing and impacting other components.

Gas transfer at crustal scales is a common driver of explosive eruptions. Two essential requirements to generate overpressures capable of driving explosive eruptions include (1) the release of volatile components from magma and (2) a means of focusing and accumulating these volatiles at a specific level or levels within the crust. The first requirement means that magma is volatile-saturated, and this must be a common or ubiquitous occurrence prior to sudden explosive eruptions (Edmonds and Woods 2018). Volatile saturation and exsolution occur at deep crustal or mantle depths when CO_2_ is involved, and at shallow levels (typically < 6 km) when water-rich magmas are involved (Dixon and Stolper 1995). The second requirement is some sort of seal to allow gas accumulation.

Magmas release gas in two ways: by ascending and undergoing decompression (first boiling) (Cashman and Blundy 2000), and by cooling and crystallizing (second boiling) (Tait et al. 1989). Sudden upward magma movement, for example, a dyke injection or an episode of magma replenishment, will trigger rapid volatile release that can supply gas to a shallow magmatic–hydrothermal system. By contrast, stationary fluid-saturated magma bodies that are cooling and crystallizing, such as sills and laccoliths, will steadily release volatiles that can accumulate within or at the top of the magma body, or leak through it to higher crustal levels.

**Fig. 7:**
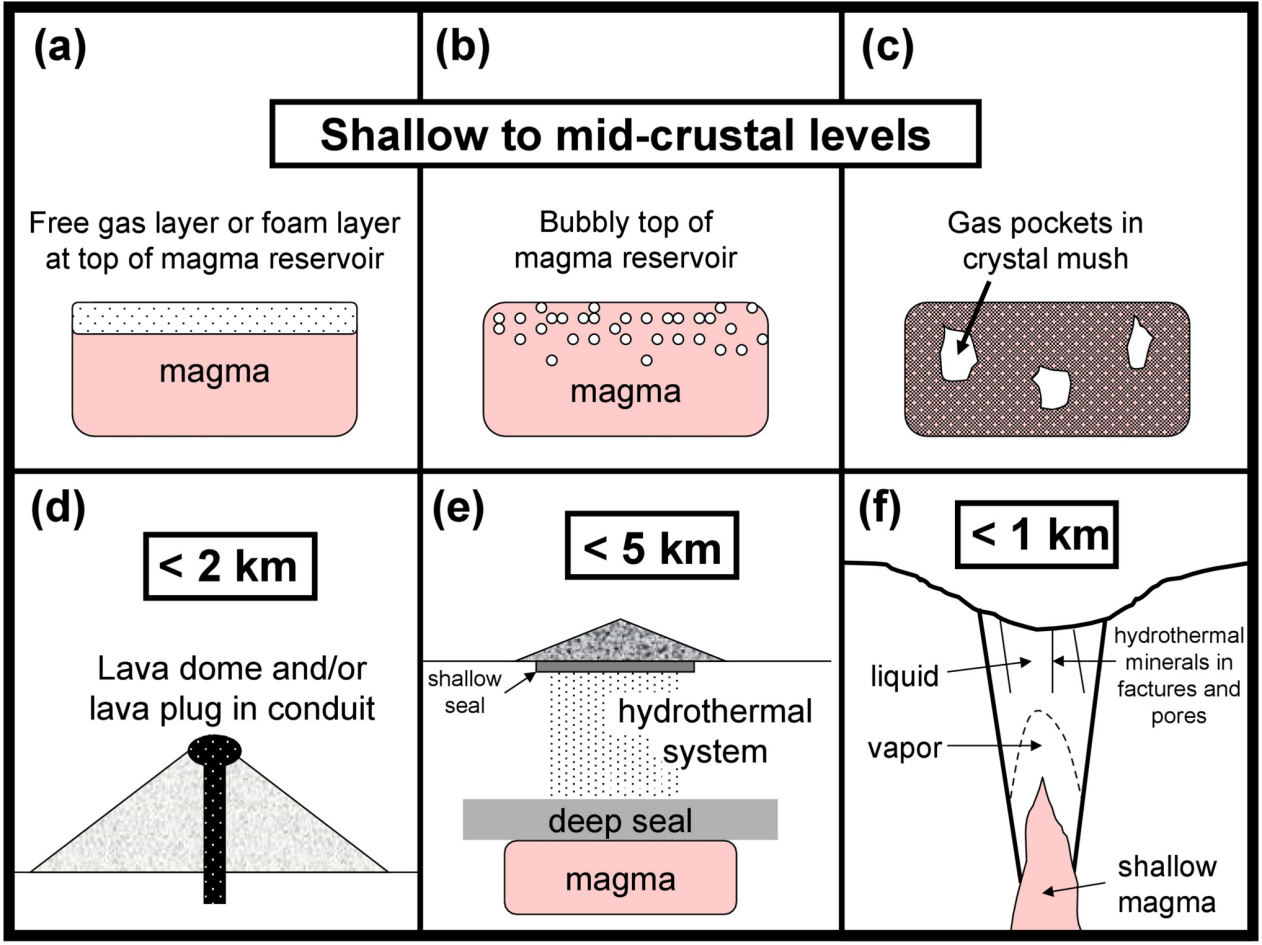
Locations and sources of overpressure. **a** A foam layer or free gas layer sitting on top of a magma body. **b** A magma body containing free gas bubbles in its upper reaches. **c** Gas pockets contained within a crystal mush. **d** A lava dome and/or lava plug filling the upper parts of a conduit system. **e** A seal separating a deeper magma body from a shallower hydrothermal system. **f** A shallow conduit system containing a magma intrusion with a hydrothermal system above. The hydrothermal system comprises a vapor zone directly above the magma, with an overlying liquid zone. Here fractures and pore spaces are filled with hydrothermal minerals, creating a seal

We propose that there are seven principal mechanisms by which gas released from magma can accumulate and pressurize. These mechanisms are shown schematically in Fig. 7. (1) Gas can accumulate in a foam layer at the top of a magma body at shallow to mid-crustal levels (Jaupart and Vergniolle 1989) (Fig. 7a). The foam can build to a point where it destabilizes, resulting in catastrophic gas release and eruption. The 3 July 2019 Stromboli eruption exemplifies this mechanism (Aiuppa et al. 2021). (2) Cooling and crystallizing magmas commonly reach volatile saturation, allowing for the development of a free gas phase distributed in the magma (Edmonds and Woods 2018). The free gas may accumulate at the top of the reservoir (Wallace et al. 1999) (Fig. 7b) or within pockets in the crystal mush (Barth et al. 2019) (Fig. 7c). Such magmas generate overpressure (Blake 1984; Tait et al. 1989), and the timing of gas saturation may serve as a trigger for an explosive eruption (Stock et al. 2016). (3) A lava dome or lava plug contained within the upper sections of a conduit system can result in gas accumulation and pressurization beneath (Stix et al. 1993; Boudon et al. 2015) (Fig. 7d). The pressurization can develop over variable timescales, as observed at Sinabung volcano in Indonesia in 2016–2017 (Kunrat et al. 2022) and at Galeras volcano in 1992–1993 (Stix et al. 1997). (4) If a lava dome is present, overpressure will be enhanced by the intrusion of volatile-bearing magma into the base of the lava dome system from deeper levels (Boudon et al. 2015). Such a configuration may have been present prior to the April 2021 explosive eruptions of Soufrière St. Vincent (Contreras-Arratia et al. 2024; Robertson et al. 2024; Sparks et al. 2024; Stinton 2024). (5) Impermeable zones are sometimes present between a deeper magmatic system and a shallower hydrothermal system (Fig. 7e). Such boundaries are present on a caldera scale at Campi Flegrei, where sills emplaced at 3–4 km depth are overlain by impermeable rocks (thermometamorphic mineral seals) that isolate the overlying hydrothermal system (Lima et al. 2021; Kilburn et al. 2023). (6) At shallow levels, magma bodies such as dykes, sills, and pods may have a hydrothermal envelope comprising vapor, brine, and mixed fluid zones where volatiles are accumulated (Fig. 7f). Such a configuration is likely present at Poás beneath the crater lake (de Moor et al. 2019) and at Whakaari as well (Christenson et al. 2017). (7) The shallow hydrothermal system may itself be sealed between its top and the surface, hence pressurized, by minerals that have precipitated in the crack system and shallow conduit (Kennedy et al. 2020; Mick et al. 2021) (Fig. 7f). These minerals can have low strengths, e.g., clays, so the seal may be weak and easily ruptured (Heap et al. 2022; Stix et al. 2024). Christenson et al. (2017) present a framework model of this sealing process, which involves the precipitation of liquid sulfur and other minerals, and variations in heat and mass flux of volatiles from depth.

This template for pressurization, as shown in Fig. 7, implies that, at times, a magmatic-hydrothermal system beneath a volcano may be simultaneously pressurized at different locations, depths, and pressures. For example, such a condition likely was present at Ontake prior to the 2014 eruption, when the shallow pressurized hydrothermal system was fed from below by magmatic fluids pressurized at deeper levels (Kato et al. 2015; Mori et al. 2016; Terakawa 2017). The template may help to identify the principal locations of pressurization under the volcano. Finally, we may be able to use the template to equate precursory signals to these different pressurization zones.

## Exportable precursors

A crucially important question to ask ourselves is the quality of our precursors, i.e., how statistically significant are they in terms of accurate forecasts? How sensitive and accurate (true alarm rate) or inaccurate (false alarm rate) are these precursors? How can we set a reasonable threshold and hence do an accurate forecast based on this threshold and a specific precursory signal or signals? How exportable are these precursors? For example, caldera-wide inflation at Hakone was small, yet a phreatic event was produced from a shallow and localized source of overpressure. What is this information telling us for Campi Flegrei in Italy, a highly active caldera system that has experienced caldera-wide deformation of ~ 4 m in 70 years? These are the critical questions that we face, and that we must try to answer.

For a number of the examples that we have examined above, a striking observation is that small changes were occurring months before the principal explosive eruption. To illustrate, CO_2_ levels increased steadily over the course of a year at Stromboli. Early subtle magmatic leaks appeared at Poás 5–6 months beforehand. Slow inflation at Hakone began occurring about one year prior. At Soufrière St. Vincent, slow and steady effusion of lava occurred for slightly longer than 3 months before the effusive-explosive transition. This common pattern of long-term and low-level activity demonstrates that these systems possess a sufficient degree of crustal interconnectivity to allow for the passage and transfer of magma and/or magmatic fluids from deeper crustal levels to shallower levels. In the case of Stromboli, the passage of CO_2_ likely began in the mantle. At Poás and Whakaari, much of this passage was likely at very shallow crustal levels, probably < 1 km, although deeper processes also may have been active yet masked by the events at shallow depths. This commonality suggests a certain degree of crustal permeability at various and variable depths. The exact nature of this permeable flow is a challenging question. It could be through fractures and faults (Sibson 1996), other pathways such as gas channels in crystal mush (Parmigiani et al. 2016), and/or filter pressing (Sisson and Bacon 1999), to illustrate several mechanisms.

Based on the examples we have presented above, we propose six types of precursory signals that could consistently serve as warnings to subsequent explosive eruptions. Not all of these are universally exportable to all volcanoes worldwide. Instead, some of these signals are specific to a certain class of volcano. Yet by judiciously using these precursors and potential thresholds associated with them, it may be possible to identify crucially important points in time when a system undergoes a significant change in its progression toward an eventual explosive eruption.

Our first type of signal is based on our experience at Stromboli, where CO_2_/SO_2_ and especially CO_2_ concentrations reveal important information prior to large explosive eruptions. To illustrate, the situation prior to the 3 July 2019 eruption was characterized by steady and significant increases in CO_2_ concentrations over months measured by the summit Multi-GAS station. The changes occurred for maximum, minimum, mean, and median concentrations (see Fig. 1e). A threshold of ~ 75 ppm for mean or median CO_2_ concentrations might be established, which could pinpoint a change from “normal” behavior to a state that might be characterized as “abnormal,” potentially serving as an accurate indicator to the proximity of a large eruption. Notably, mean CO_2_ concentrations at Stromboli again reached this threshold before the 20 July 2020 major explosion, the largest of this class of events on record (Voloschina et al. 2023; Fig. 1e). Such specific thresholds may not be directly exportable to other open-vent basaltic systems, as they depend on the distance between the source (the active vents) and measurement sites. However, the observation of combined CO_2_ concentration and CO_2_/SO_2_ ratio increases should be taken as general evidence of the magmatic system being flushed by excess CO_2_-rich gas, potentially sourced by buoyant mafic magma at depth. Increasing CO_2_ fluxes (Fig. 1c) may reveal critical clues in establishing that the gas change is volumetrically important, i.e., that a larger than normal volume of magma is degassing CO_2_ at depth, and is hence primed to erupt. It is also important to consider that the CO_2_ concentration/flux increase will only be observable if the source area (the gas accumulation zone) is deep in the system; shallow gas accumulation may produce weak (or even no) signals in the gas record.

Second, transitions between hydrothermally dominated and magmatically dominated activity could provide useful warnings. At Poás, changes in SO_2_/CO_2_ and H_2_S/SO_2_ were observed at two different timescales, over months and also over weeks to days. The data at both timescales could be used to develop thresholds between hydrothermal and magmatic behavior, e.g., SO_2_/CO_2_ of ~ 0.1 and H_2_S/SO_2_ of 1–2 (see Fig. 2). During a period of dominantly hydrothermal activity, the interface between the magmatic system and the hydrothermal system may be sufficiently strong to allow for substantial overpressure to develop at some depth, increasing the likelihood of an explosive eruption. At Poás, the VT swarm on 26 March 2019 may have been generated by this overpressure when it exceeded the tensile strength of the rocks forming the seal, allowing a pathway to be established to the surface for magmatic fluids and magma. At Poás, a SO_2_/CO_2_ ratio of 1.5 is being used as the threshold above which magmatic gases are considered to be reaching the shallow hydrothermal system at a rate sufficient to generate eruptions. Ongoing work suggests that this ratio shows some evidence of being proportional to eruption column height. Similar changes from hydrothermal to magmatic behavior were revealed at Hakone, where the earthquake swarm of May 2015 coincided with substantial increases in CO_2_/H_2_S and CO_2_/CH_4_ (Ohba et al. 2019), indicating that magmatic fluids were able to exploit new pathways to the surface 1–2 months ahead of the phreatic eruption of 29–30 June. Hence, thresholds that use these gas ratios may be exportable to other volcanoes with complex magmatic-hydrothermal plumbing.

Third, volcanotectonic (VT) seismic swarms can indicate the opening of a conduit/crack system. At Poás, the VT seismic swarm of 26 March 2017 clearly jumpstarted the eruptive sequence starting in April (Fig. 2d). The swarm reflected the establishment of a crack or conduit from magma residing at shallow levels to the surface. At Hakone, the May 2015 earthquake swarm in May provided a pathway for magmatic fluids to reach the surface, followed by the late June eruption. Similarly, at Soufrière St. Vincent, two VT swarms were observed on 23–24 March and 5–6 April 2021, soon before the explosive eruptions began on 9 April (Joseph et al. 2022). The second swarm coincided with a large acceleration in the effusion rate of the lava dome (Dualeh et al. 2023; Stinton 2024), followed soon after by the explosive emission of volatile-rich magma (Contreras-Arratia et al. 2024). These examples show that such earthquake swarms can be highly significant indicators of impending explosive eruptions if conduit systems are able to reach the surface. We fully acknowledge that VT seismic swarms, with no associated eruption, are common occurrences at active volcanoes. Nevertheless, they should be carefully monitored for the possibility that such swarms are revealing the establishment of a conduit or crack system that could permit gases and/or gas-rich magma to reach the surface.

Fourth, the nature, spatial scale, and degree of ground deformation at caldera volcanoes could provide critical clues to an impending explosive eruption. At Hakone, low levels of caldera-scale inflation were observed starting about a year before the June 2015 eruption. By contrast, a highly localized circular inflation pattern < 500 m in diameter, measured by InSAR, began to be observed 6–7 months beforehand (Fig. 5). This spatial “focusing” of inflation may be significant in three respects. (1) It may be an indication that the pressure source is shallowing. (2) It may be an indication of proximity to an explosive eruption. (3) It may allow us to pinpoint the location of eruptive vents in advance. Such observations may be exportable to all active caldera systems that exhibit ground movements.

Fifth, banded tremor is highly significant for its relationship to explosive eruptions. Such seismic signals have been observed before such events at Whakaari (Stix et al. 2024) (Fig. 3), Soufrière St. Vincent (Joseph et al. 2022) (Fig. 6), Soufrière Hills on Montserrat (Stix 2018), Mount Etna in Italy (Gresta et al. 1996), Karkar Papua New Guinea (McKee et al. 1981), and Nevado del Ruiz Colombia (Martinelli 1990). Banded tremor is a reliable indicator that the subsurface plumbing system beneath a volcano is undergoing pressurization (Cannata et al. 2010; Caudron et al. 2022b). The nature, timescales, and durations of the tremor can vary among different systems, suggesting that a number of processes may be involved. In some cases, for example, repetitive low frequency “drumbeat” earthquakes sometimes occurring with tremor have been documented with typical timescales of seconds to minutes, and appear to be associated with magma ascent processes coupled with gas release processes (Pallister et al. 2013; Bell et al. 2017; Ichihara et al. 2023). In our view, the recognition of a banded tremor pattern in real time should be a crucial clue that a system is overpressurized and has the potential to erupt explosively. Banded tremor may occur without an ensuing eruption, but in such cases, the system may be close or very close to its eruptive threshold. Such “failed” eruptions should thus be expected at times, and such occurrences should not reduce or minimize the significance and utility of this very interesting and important seismic signal. These observations are exportable to volcanoes where subsurface magmatic-hydrothermal interactions are occurring.

Sixth, Caudron et al. (2019) have recently developed the Displacement Seismic Amplitude Ratio (DSAR), which is essentially a ratio of volcanic tremor amplitudes at 4.5–8 Hz compared to those at 8–16 Hz. These authors have shown that DSAR increases steadily before a number of eruptions on timescales of months to years, then decreases after the eruption over comparable timescales. Ardid et al. (2022) extended this analysis by considering the nDSAR rate variance over shorter timescales. They found that this parameter reaches a maximum value from 1 to 3 weeks before explosive eruptions at a number of volcanoes, including the 2019 Whakaari eruption. These interesting findings are important because they appear to be exportable, and they can be used at multiple timescales from years to weeks and possibly even days. The increases in DSAR and nDSAR rate variance are driven by the increasing amplitudes of the lower frequencies, which likely reflect a system undergoing pressurization. The use of these measures, together with other precursors such as banded tremor, could greatly aid in accurate forecasting efforts in the near future.

Finally, Ontake provides a useful and cautionary example of a system that is geophysically quiet. Very low levels of activity were observed at the volcano prior to both the 2007 and 2014 eruptions. In such situations, any sudden increase in activity, even if small, may be an indication that the system has started to pressurize above baseline values. The abrupt increase in VT and LP seismicity beginning on or about 6 September 2014, three weeks before the eruption, may reflect the initiation of such a pressurization event.

## Using and applying thresholds and transitions

As illustrated by the examples discussed herein, precursory signals have variable timescales over several orders of magnitude. A significant challenge is to recognize these different precursors and their associated timescales in real time. An important point from our examples is that the development of conditions leading to a significant explosive eruption can be long, typically months to a year or more. This window provides us with a potential opportunity to identify key points in time in terms of hazard and risk. Hence, we now explore the utility of thresholds, which could help identify these significant points from an unrest and forecasting perspective (Kilburn and Bell 2022).

We begin with three illustrations of potential late-stage thresholds, which hold impactful information. At Stromboli, mean CO_2_ concentrations at the summit MultiGAS station have reached 100 ppm on a regular basis. At Poás, H_2_S/SO_2_ is approaching zero and SO_2_/CO_2_ is increasing well above the threshold value of 1.5. At Whakaari, banded tremor is occurring at decreasing intervals. These data all indicate that the volcanoes have a heightened probability of erupting soon in an explosive fashion, with potentially significant impacts on the surrounding area. In these circumstances, a clear and simple warning could be communicated.

Now let us go back in time and consider scenarios and conditions that are intermediate and less threatening. Mean CO_2_ concentrations Stromboli are regularly reaching 75 ppm. The SO_2_/CO_2_ ratio at Poás is varying between 1 and 4 and generally increasing. At Whakaari, the tremor signal is present and generally increasing in amplitude with time. Such data and potential thresholds are more difficult to interpret and understand, yet this type of information also holds promise in terms of informing the public of potential danger levels.

Finally, let us go to the earliest stages of activity, where indicators and precursors are at low levels and difficult to recognize. Alert levels may be at 0–2, and the volcano system is considered comparatively quiet, resting, etc. At Stromboli, mean CO_2_ concentrations are hovering around 50 ppm. At Poás, SO_2_/CO_2_ is somewhat variable, with a mean value of < 1 and no obvious temporal trend. Tremor is absent or sporadic at Whakaari. At this stage, there may be value in conveying various “what if” scenarios. What if mean CO_2_ increased to > 50 ppm? What if H_2_S/SO_2_ started declining? What if the occurrence and amplitude of tremor began increasing? For systems prone to sudden explosive eruptions, it is important to reinforce that even though activity seems to be at low levels, these periods of comparatively low-level activity may be reflecting the accumulation of gas and pressure within the plumbing system. It is crucial to highlight the need for continuous monitoring, vigilance, and preparedness because changes can occur rapidly prior to the violent release of accumulated gases. However, with no particular trends in the data, it may be appropriate to communicate that the probability of activity remaining low or increasing over moderate timeframes is roughly the same.

At Soufrière St. Vincent, the lava dome grew slowly at a constant rate from late December 2020 to early April 2021. To people living on the coast, there was no impact and effectively no eruption. Yet an eruption was indeed occurring, and scientists impressed this point upon the public (Joseph et al. 2022). Furthermore, based on previous unrest at this system, they emphasized the possibility that the eruption could change from effusive to explosive, potentially on short timescales. Additionally, the older population retained memory of the 1979 explosive activity. On 8 April 2021, when the lava dome began to show visible signs of accelerated growth and banded tremor was recognized in real time (Fig. 6), scientists, civil defense, and the public were all in a good position to take effective action, e.g., evacuating from vulnerable locations. This example well illustrates an ideal-case scenario of an important and very fast transition in volcano behavior. Anticipating such transitions is a key challenge moving forward. A further challenge is when such transitions take place at a late stage, sometimes quickly, with little time left before the volcano erupts. At this stage, the system is overpressured, with a high probability of erupting explosively.

## New ways forward

The four key pieces of information needed for a full forecast are timing, location, size, and style of the eruption (Acocella et al. 2024). Timing tells us when the eruption will occur. Location tells us where it will occur; this is particularly important for caldera systems that are large spatially with multiple vents. Size tells us the magnitude of the eruption and the extent of the area affected. Style tells us whether an eruption will be effusive, phreatic, magmatic, or something in between, e.g., phreatomagmatic. These four parameters are extremely difficult to determine prior to an eruption, and this difficulty constitutes a major challenge for volcanologists during the next decade. Even determining when an eruption will occur is highly problematic, as clearly shown by the examples discussed in this paper. So while we can currently anticipate increases in volcanic activity under certain circumstances, a full forecast including all four elements is not currently feasible for sudden explosive eruptions.

Yet the examples shown here reveal some useful insights for future forecasting. In terms of when an eruption occurs, the banded tremor analysis at Whakaari shows that such an approach can provide a highly accurate determination of when a phreatic eruption will occur. Such a determination can be made several days to several weeks beforehand, with potential accuracies on the order of hours. The uncertainties in such forecasts are about 1–3 days and possibly better. Regarding where an eruption might occur, the InSAR analysis at Hakone provides a remarkable level of spatial resolution, on the order of tens of meters, which revealed highly localized asymmetric inflation, with the eruptive vents corresponding closely to the zone of maximum inflation (Fig. 5). Regarding the size of an eruption, it has been suggested that the level of VT earthquake activity is a good predictor of the newly intruded volume of magma (White and McCausland 2016). The VT seismicity typically occurs at some distance from the volcano’s summit along faults, and the peak in seismicity generally occurs at the time of the initial eruption. Such information, especially when analyzed in real time, can provide a first-order estimate of the potential eruption size. At Poás, ongoing work is revealing that both the value of SO_2_/CO_2_ and the rate of increase in SO_2_/CO_2_ appear to correlate with eruption magnitude. Finally, regarding eruption style, events at Merapi volcano (Indonesia) in 2010 and at Soufrière St. Vincent in 2021 clearly demonstrate that accelerations in lava dome growth precede explosive activity, as documented by Synthetic Aperture Radar (Pallister et al. 2013; Dualeh et al. 2023). In both cases, the accelerations were rapid and occurred only days to hours before the explosive eruptions commenced. In the case of Merapi, the SAR analysis was done in near real time, providing a key element in issuing warnings to the population. At Soufrière St. Vincent, the SAR analysis was done retrospectively, and the occurrence of banded tremor was the principal warning sign that explosive activity was imminent (Fig. 6).

Simple approaches for forecasting are essential. They should be low-cost, and the data should be easy to analyze in real time prior to an explosive eruption. This is critically important when the time between the first occurrence of signals and the eruption is short (hours to days). For example, banded tremor at Whakaari, as occurred before the 2016 and 2019 eruptions, can be recorded by a single seismometer and analyzed in real time, and these analyses could be re-evaluated and re-calculated on a continual basis, either manually or in certain cases automatically. At Stromboli the gas data collected by a single MultiGAS station point to the possibility of establishing CO_2_ concentration and CO_2_/SO_2_ ratio thresholds, above which the likelihood and proximity to a paroxysm or major explosion increase. The SO_2_/CO_2_ and H_2_S/SO_2_ ratios measured at 1–2 MultiGAS stations on Poás afford a powerful means for recognizing rapid changes from hydrothermal to magmatic conditions, with the possibility or probability of explosive activity involving magma soon after such changes. Lastly, the use of VT seismicity to estimate, in real time, the potential size of an explosive eruption has both great utility and a high degree of simplicity, requiring a small number of seismometers and rapid analysis of the largest VT events for an accurate estimate of potential size.

## Conclusions and recommendations

The recent review by Acocella et al. (2024) makes an important point regarding validated vs. potential precursors. Validated precursors are those that have been tested in foresight, i.e., prior to an eruption, whereas potential precursors are those recognized in hindsight. Potential precursors may not be accurate signals of an impending eruption. They could simply indicate general unrest. They could also indicate a system that came close to erupting but did not, e.g., a failed eruption. The concept of failed eruptions is important (Moran et al. 2011; Werner et al. 2011; Falsaperla et al. 2014), because in some cases, failed eruptions must come very close to the brink of erupting. In such cases, advance warning is appropriate and needed.

Validated and exportable precursors are essential for us to move forward. Multiparametric monitoring records are short at most volcanoes worldwide, and testing potential precursors should be a fundamental goal of both research and monitoring programs. In our opinion, such precursors could include CO_2_ and CO_2_/SO_2_ thresholds demonstrating deep gas and magma influx, very low H_2_S/SO_2_ ratios signaling magmatic input and throughput, volcanotectonic seismic swarms indicating crack or conduit opening, localized or displaced inflation showing focusing of magma or fluids, and banded tremor revealing pressurization and proximity to explosive eruption. Events at Merapi and Soufrière St. Vincent demonstrate that sudden, rapid accelerations in dome growth are also reliable precursors for impending explosive eruptions. Hence, thresholds in dome growth could be established.

For explosive eruptions such as those described in this paper, volcanologists have three grand challenges to address during the next ten years. (1) We should aspire toward the ability to make full and complete forecasts of explosive eruptions, including when, where, how big, and what type. (2) A full and detailed picture of subsurface volcano plumbing, from the surface to the Moho, is needed to advance our understanding of how magmatic and hydrothermal fluids are transferred within the crust. In particular, understanding and mapping the subsurface pathways that transport these fluids is a worthy objective. The ability to identify when a batch of magma rises from a deeper zone of storage to a shallower storage zone (or the surface) is crucially important for forecasting purposes. This will require integrated geologic, geochemical, and geophysical approaches; hence, a fully integrated series of research programs on key volcanoes. (3) To properly compare data among different volcanoes, monitoring networks should collect data in a comprehensive and systematic fashion. A template comprising seismic, deformation, and gas information could be designed to achieve this goal. The networks need to be dense with precise measurements and real-time processing, and supplemented with artificial intelligence and machine learning algorithms. A primary objective should be to detect and understand subtle precursory signals which might otherwise be missed. This enhanced detection will allow us to observe highly localized changes that may be occurring at shallow levels, as well as changes that may be occurring in the deeper parts of the plumbing system. To further advance this crucially important goal, we believe the following points are worthy of consideration:

1. Because many of the precursory signals prior to these types of eruptions are small and subtle and localized around the vent, we need more instruments at or close to the vent. A key issue is how to deploy and service these instruments safely.
2. It is desirable that fully autonomous in-situ gas sensing units (e.g., MultiGAS) with real-time data streaming and processing are put in place at as many degassing volcanoes as possible, especially those with vulnerable populations nearby. As this goal is unlikely to be reached for remote or poorly accessible volcanoes, improving the current spatial and temporal resolutions of space-borne gas measurements (e.g., TROPOMI for SO_2_) should be exploited to the fullest extent possible, and satellite research should prioritize extending observations to other key gas targets (e.g., CO_2_).
3. Real-time and near real-time methods should be sought to identify and measure ground deformation which is spatially localized and either missed or poorly expressed by GNSS networks. Current techniques such as InSAR, synthetic aperture radar backscatter, and LiDAR/photogrammetry surveys by drone can be employed for this purpose, and new approaches should be developed.
4. Very long period (VLP) seismic events may hold valuable information. Such signals from Ontake reveal a shallow overpressured system capable of forming subsurface cracks which can propagate to the surface, thus initiating an eruption (Nakamichi et al. 2009; Maeda et al. 2015). At basaltic systems such as Stromboli, high levels of VLP activity appear to reflect input of CO_2_-rich, gas-charged magma into the shallow plumbing system (Giudicepietro et al. 2020).

The challenges and recommendations outlined above are both formidable and exciting. The issues and problems associated with sudden explosive eruptions provide opportunities in terms of new research and monitoring approaches, which hold potential for our community to understand, forecast, and predict explosive volcano behavior.

## Data Availability

There are no datasets associated with this paper. All data presented in the paper come from previous publications cited in the paper, figure captions, and bibliography.
